# Lo Re G, Argo A, Midiri M, Cattaneo C, eds. Radiology in Forensic Medicine: from Identification to Post-mortem Imaging: Springer; 2020. 301 pages. ISBN 978-3-319-96736-3 (hardcover), price 117.69 €; ISBN 978-3-319-96737-0 (e-Book), price 91.62 €

**DOI:** 10.3325/cmj.2020.61.73

**Published:** 2020-02

**Authors:** Vedrana Petrovečki

**The Field of Medicine:** Forensic medicine, (forensic) radiology

**Format:** Hardcover book, e-book

**Audience:** Although the target audience are forensic medicine specialists, the book will also be of interest to radiology specialists, especially those dealing with injuries from physical conflicts, gunshot and stab wounds, and injuries from road traffic accidents.

**Purpose:** In the last 50 years, we have witnessed a rapid progress in radiology. Not much time has passed since the introduction of computed tomography (CT), which soon advanced to multi-slice computed tomography (MSCT), and the introduction of magnetic resonance (MR) imaging. The book stresses the usefulness of these new radiological techniques, in addition to conventional radiology, in forensic medicine, referring to them as postmortem CT (PMCT) or postmortem MR (PMMR). It also presents angiographic approaches as a useful tool for the identification of vascular injuries. The increased use of radiological techniques in forensic medicine led to the introduction of a new forensic discipline – forensic radiology –and created the need for guidelines regarding the use of different methods.

The book introduces the term “virtopsy” (a combination of “virtual” and “autopsy”), which is particularly useful because the stored data are available in the circumstances when a second opinion is needed. However, despite the advances in radiology, forensic autopsy remains the “gold standard” in forensic diagnostics of an unknown cause of death. This irreplaceable technique for determining the cause of death, and the only accepted technique in court proceedings, can only be improved but not substituted by radiological methods.

**Content:** The book consists of 29 chapters, and begins with the history of forensic radiology, describing one of the first applications of forensic radiology. At the Natural History Museum in Vienna in 1896, classical x-rays processing showed that the analyzed mummy considered as human was in fact a large bird. The second chapter discusses the importance and the limitations of classical forensic medicine, in particular forensic autopsy.

The following chapters cover specific aspects of the field. Forensic radiology is emphasized to have important potential applications in court proceedings in demonstrating physical abuse of children, women, and the elderly; diagnosing drug addiction; identifying drugs smuggling; or determining the trajectory of stab and gunshot wounds in living persons and during post-mortem analysis. Radiological techniques related to the detection of drugs are additionally covered in a separate chapter. The role of forensic radiology in the age estimation of deceased unidentified persons is covered in two separate chapters: the first dealing with the identification according to dental or skeletal morphology and the second discussing the recognition of post-mortem changes under different circumstances (asphyxia, road accidents, physical abuse, firearm injuries). A few chapters are dedicated to particular types of injuries, presenting them in a region-specific manner and in relation to postmortem changes, emphasizing the requirement for far thinner layers than in standard PMCT (microradiology). Specialized chapters describe the radiological signs of drowning and radiological approaches in abrupt adult and fetal death, and their ability to distinguish post-mortem from pre-existing injuries, potentially related to the cause of death. The post-mortem analysis of the skull and brain is outlined in a separate chapter, containing the PMCT brain protocol, description of postmortem changes in the brain tissue, various forms of intracranial bleeding, and occult injuries that can be easily overlooked by standard forensic autopsy. The applications of radiological techniques in archeology and in occupational diseases (pneumoconiosis) are given a chapter each. There is also a chapter dealing with the ability of radiological techniques to determine whether a person is telling the truth or not. An especially valuable chapter covers mass disasters, offering a basic perspective on the organization of the identification team and emphasizing the role of mobile x-ray and CT devices, and radiologists' contribution to the identification of separate body parts, dangerous foreign bodies, personal metal objects, and characteristics related to a person's identity.

Special chapters are dedicated to methodology, indications, standard protocols, and procedures in conventional radiology and PMCT. There is also one chapter dealing with the contribution of forensic radiology to the publication practices in forensic science, which emphasizes its importance in court proceedings and addresses radiology malpractice.

The book concludes with a chapter on the bioethical aspects of post-mortem radiological analysis. It discusses the situations when classical autopsy is inevitable, such in court proceedings, as well as the cases when virtopsy could give satisfactory answers, avoiding not only physical intrusion into the body of the deceased but also moral intrusion into the rights of family and friends who oppose the autopsy.

**The highlights:** The book contains numerous high quality images that successfully illustrate all the aspects of a chapter. We should also mention the extensiveness of the literature at the end of each chapter. References can help the interested readers to continue deepening their knowledge according to their personal interests.

**Commentary:** There is some redundancy across chapters and slight inconsistencies in citing styles. These are not unexpected, especially considering the number of chapters and the individual contributors, and certainly do not diminish the book’s value.

